# Hard-working or hardly working dogs stay young longer? Lifetime sports engagement, joint activity with the owner and breed type are associated with the severity of canine cognitive decline

**DOI:** 10.3389/fvets.2026.1833531

**Published:** 2026-04-28

**Authors:** Csenge Anna Lugosi, Petra Dobos, Péter Pongrácz

**Affiliations:** Department of Ethology, ELTE Eötvös Loránd University, Budapest, Hungary

**Keywords:** activity level, breed types, canine cognitive decline, dog sports, mixed breeds

## Abstract

**Introduction:**

Companion dogs normally reach >7–8 years of age, which is enough to develop neuro-degenerative cognitive decline. There is a vivid interest in the factors that can accelerate canine cognitive decline (CCD), or on the contrary, act as a preventive measure. We investigated the potential effect of several factors on dogs’ CCD scores: functional breed type (cooperative vs. independent working breeds), lifetime sports activity, activities with the owner, and priorities of the owner when choosing a dog.

**Methods:**

The internationally distributed questionnaire was completed for *N* = 858 senior dogs (>7 years). We collected data about the dogs’ activity levels, regular sport/working engagement, and body condition. Owners described their dogs’ behavior regarding the signs of cognitive decline. Owners rated also the importance of aspects that could influence their choices at the time of their dog’s acquisition.

**Results:**

We found that a lifetime sports career (*p* < 0.001) and joint activities with the owner (*p* = 0.037) had the strongest negative association with CCD. Dogs that were considered as sports companions, regardless of the breed type, had lower CCD scores (in other words, they were in better mental condition according to the owners) than dogs who were regarded as ‘domestic’ or ‘breeding’ animals (*p* = 0.041). Dogs that have not been selected for working tasks (toy breeds and mixed breeds) benefited the most from joint activities with their owner, while cooperative and independent working breeds had lower CCD scores even if they only sporadically participated in physical exercise. Those dogs with owners that strongly prioritized either health and sound behavior (*p* = 0.042), or high breeding quality (*p* = 0.004), had low CCD scores. When owners showed preference for fashionable or rare breeds, this did not affect the dog’s cognitive decline (*p* = 0.830).

**Conclusion:**

With conscientious choices, and opting for an active lifestyle with their dogs, owners can slow down the onset and severity of CCD. According to our results, working breed types that have been selected for a stronger drive for sports activities may also benefit from physical exercise. Generally long-living toy breeds and mixed breeds would be especially important to involve in joint activities with the owner to avoid severe CCD.

## 1 Introduction

Aging, as a complex, unidirectional process, is characterized by a progressive loss of physiological integrity, leading to impaired function and increased vulnerability to death (1). Mortality rate across lifespan, forms a J-shaped curve in most mammalian populations: the high early age mortality declines to a minimum (*q*_min_) nearing the approach of adulthood, followed by exponential acceleration of mortality at midlife in association with increased chronic degenerative disease and dysfunction that collectively defines senescence (2). Similar to humans, animal brains are also susceptible to aging and suffer irreversible molecular, structural, and functional changes that can lead to cognitive decline and neurodegeneration (3). Cognitive functions, defined here to include perception, memory, learning, problem-solving, and innovation, play an essential role in the ability to process and respond to environmental information (4). Some cognitive deterioration may also occur in a few wild-living species, mainly in the case of highly social animals with longer lifespans and complex nervous systems [e.g., whales, elephants, non-human primates (5)]. However, impaired cognitive performance (along with physical decline) reduces the capacity of wild animals to respond appropriately to environmental challenges and makes them more vulnerable to survive the onset of old age (6).

Contrary to a life in the wild, where predators, physical hardships, or failure to effectively forage, prevents most species from living long enough to show age-related decline, life in a buffered human environment (e.g., wild species in zoo parks, or exotic and domesticated animals kept as companions), allows many animals to reach old age (7, 8). Typically, domestic dogs and cats reach 7–8 years of age, with an increasing prevalence of developing cognitive dysfunction, and because we provide them with safe environments and veterinary care, they often survive well beyond the onset of senescence (9).

Just as in humans, cognitive decline is considered to be a natural part of the biological aging process in dogs, and also similar to humans, due to severe and irreversible brain atrophy (10), a subset of dogs will develop dementia in old age, which is referred to as Canine Cognitive Dysfunction (CCD) (11). A decline in the physical or mental health of older dogs can be a challenge for the owners, when their relationship with their dog is compromised by the cognitive and behavioral changes that occur (12). Although dog owners tend to consider many physiological and behavioral changes in old dogs to be part of the normal aging process, it is important to differentiate between normal aging and pathologic aging, since behavioral changes may be the first indication of declining health and welfare in old dogs. Normal aging is defined as gradual, age-related slowing of cognitive processes without major impairment of daily functioning, while CCD is a syndrome that mirrors many of the clinical features of human Alzheimer’s Disease (AD). CCD includes a progressive loss of cognitive function such as decreased ability to learn, and impairments of memory, furthermore, it can be characterized by increased anxiety, loss of normal sleep patterns, aimless wandering, and dysfunction with interactions and housetraining (13). Importantly, the diagnosis of CCD should only be made when all other causes of behavioral changes, including those related to sensory decline (particularly hearing and vision), environmental effects, pain, degenerative diseases, and other geriatric changes, or underlying disease processes, have been ruled out (14).

CCD can be reliably diagnosed by using the owner or veterinarian administered Canine Cognitive Dysfunction Rating Scale (CCDRS) (15). This tool is based on a validated questionnaire that assesses the dog’s cognitive functions by using 13 variables scored on a scale of 1–5 points each, leading to CCD scores that can range from 13 to 65 points. CCD scores of 50 points and above are indicative of a diagnosis of CCD.

Besides the discovery and description of behavioral signs of cognitive aging (15), there is a vivid interest in the potential factors that could influence the development of CCD. The risk factors and causes of CCD in dogs have not been fully investigated, but similar to human dementia, age, gender, oxidative stress, and deficiency of sex hormones (testosterone and estrogens) appears to be associated with increased risk of accelerated brain aging and CCD in dogs. Age has also been reported to be a risk factor for AD in people (16). In dogs, the prevalence of CCD naturally increases with advancing age, so that eventually >50% of dogs above 15 years old are affected (14). In people, the incidence of AD in females is almost twice of that in male subjects (17), and similarly the incidence of CCD in female dogs was more than twice that of male dogs (18). Sex hormone deficiency may be a risk factor for brain aging and dementia in people (19) and dogs (18). Oxidative stress may also be a contributing factor for brain aging in humans (20) and dogs (21) too.

There were indications that some of the risk factors can be eliminated or mitigated by nutritional interventions both in the case of humans (22, 23) and dogs (10, 24). As owners’ attitude about dogs can have a great influence on the dog’s overall quality of life (activity, nutrition, veterinary care) [e.g.: (25, 26)], we can assume that an owners’ attitude can also influence dogs’ cognitive aging. In the case of humans, physical activity has been identified as one of the main modifiable factors that reduces the risk of developing dementia (from all causes), and Alzheimer’s disease (27, 28), and it also improves the performance of subjects with established dementia (29). Since brain aging in people and dogs shares many similarities (30), it is reasonable to assume that the effects of regular sport activity on cognitive decline in people, may also apply to dogs.

Based on the 10 thousand+ database of canine subjects in the Dog Aging Project (31), physical activity showed a robust negative association with the deleterious signs of canine cognitive dysfunction (CDD). This result was naturally a correlative one, therefore further investigations are warranted to discover more about the potential causative effects of physical activity on cognitive aging in dogs. In a recent empirical study, it was found that even a short, 3-month course of complex physical and cognitive training and exercises, had a positive effect on older dogs’ social behavior, neophilia, and problem-solving behavior (32). The authors noted that the effect of this type of intervention can be positively influenced by an earlier start and longer application during the lifetime of the dog. Some authors highlighted that there was a need for a practical, easy to use instrument/test battery, that could be used for actively working (‘utility’) dogs’ assessment, with regard to their age-related cognitive decline (33). It was also recognized that different physical capacities would be necessary to be retained (therefore also assessed), along with aging, in the case of working dogs, compared to average companion dogs (34).

However, to our best knowledge there have not been any publications which tested for the association between lifetime work-related activity and changes in cognitive/behavioral performance of older dogs. Prompted by the mainly positive results of investigations on the protecting effect of exercise and physical activity against dementia and AD in humans (27–29), our project targets this gap by investigating the potential differences of cognitive performance between aged dogs, in connection with their lifetime activity engagement, and functional selection past.

Previous studies showed contradictory results regarding the association of CCD with dog breeds. Salvin et al. (35) found that the prevalence of cognitive decline does not differ according to typical breed size, even though large breed dogs have a shorter lifespan than small breed dogs (35). Other studies reported that small breeds had greater odds of showing age-related cognitive impairment than medium or large sized dogs, although weight was not a statistically significant predictor variable (18). Katina et al. (24) observed greater prevalence of CCD in medium/large sized breeds when compared to small sized breeds in the age group of 11–13 years, while no difference was observed in the age group of 8–11 years, and weight had no effect. Kraus et al. (36) demonstrated that while there was no clear correlation between body size and the onset of senescence, there was a strong positive relationship between size and aging rate, thus, they concluded that dogs of large breeds died younger than small breed dogs, mainly because they aged more quickly. However, it was never investigated whether the breeds’ original function could influence dogs’ cognitive aging.

Functional breed selection in the more recent (post-domestication) history of many dog breeds offers a successful lead for the investigation of behavioral phenotypes that relate to dog–human interactions (37). This approach does not focus on the specific working task of the dogs, but concentrates on how they are supposed to perform in a broader sense of dog–human interactions (38). There are two main types of working dogs from the aspect of their function: dogs that work in cooperation with, or independently from, their human partner (39). For cooperative dogs, it was adaptive to pay attention to regular visual and acoustic cueing from their handler and execute their instructions without hesitation. In the case of independent working dogs, they were selected to make decisions and solve problems on their own during their work. It was found that the two types of working dogs performed equally well when the task did not involve human contribution, however, cooperative breeds were more successful when they had to rely on human communicative signals, and they are more dependent on human help (40, 41).

In this paper, we hypothesize that breed function may show association with the mitigating effect of work/sports-related activity on age-induced cognitive decline. We predict that breeds that were selected for well-identifiable work tasks will show a stronger benefit of lifetime sports/work-related activities than dog breeds that had no clear work function (i.e., the so-called companion or ‘toy’ dog breeds), which can be further supported by the findings of earlier research that showed longer life expectancy and more prolonged exposure to brain aging in small-sized toy dogs (18). Furthermore, we hypothesize that work-related lifetime engagement is associated with the speed of age-induced cognitive decline. We predict that dogs that were involved in regular work- or sports activity for most of their lives will show less signs of old-age cognitive decline, than dogs with no regular sports/ working activity during their life.

Our goal was to comprehensively investigate potential effects that could influence dogs’ cognitive decline through a large-scale questionnaire survey, which included questions about the dogs’ demography, breed group, health, aging, activity, and the owners’ attitude about the dog.

## 2 Methods

### 2.1 Ethics approval and consent to participate

The task for the participants was to provide information about their aged dogs’ demographic descriptors, behavior, and details of their dogs’ sport-related activity. In this study, we did not collect any personal data about the human respondents that would reveal their identity. The respondents took part in the research voluntarily and were asked for their informed consent and agreement to participate in a scientific study, as well as to acknowledge that their responses will be used for scientific purposes only. We communicated with participants via email, and we permanently deleted the emails after extracting the necessary data. This type of research does not require any further or specific human ethics approval in Hungary.

### 2.2 The questionnaire

We distributed two identical variants of the questionnaire: one was in English and the other in Hungarian language. The English version of the questionnaire can be seen in the Supplementary materials. The online questionnaire started with an (i) introduction/ informed consent; then we asked for the (ii) basic demographic details of the dog owner, including their type of locality and residence where they kept the dog. The next (iii) section was about the demographic description of the dog, including the details of the conditions of housing and potential other dogs in the household. Then (iv) we asked questions about the importance of the various reasons why the owner originally chose the dog, and there were also detailed questions about their joint activities with the dog, including its potential sports/working career. The following sections (v) asked about the body condition of the dog, plus we had questions whether the dog had any serious accidents and/or health conditions that potentially threatened or stopped its sports/working career. The last section (vi) of the questionnaire contained questions about the signs of canine cognitive aging, which were adopted from the original work of Salvin et al. (15).

Before launching the survey to the public, a small-scale test run was conducted to ensure that the questions and answers were clearly understandable for respondents and the platform works smoothly. Colleagues and students (*N* = 8) from the Department of Ethology completed the survey, and after amendments based on their feedback, the items were finalized. The responses from the test run were not included to the final data set.

It took approximately 15 min to complete the survey. We distributed the questionnaire among various social media (Facebook) groups with the ‘snowball method’. This method starts with a convenience sample where respondents are also encouraged to spread the questionnaire among their acquaintances. Besides social media surfaces of the involved institutes (Department of Ethology and the “Family Dog Project” at the Eötvös Loránd University, Budapest, Hungary), we also targeted specific organizations and communities through their social media (e.g., Morris Animal Foundation, USA; American Kennel Club; various international purebred and working dog fanciers’ groups) where dog owners, trainers and enthusiasts were likely to be accessible. The questionnaire was available from May 2025 until October 2025.

### 2.3 Participants

We asked dog owners to complete the questionnaire if they had a dog older than 7 years. First, we provided a brief descriptive report about the main characteristics of our canine subjects. We welcomed the data of purebred and mixed-breed dogs as well. In this study, we did not differentiate between breed-crosses (such as, for example, the Cavapoo) and ‘mongrels’ (mixed-breeds without breed-identifiable parents). Non-purebred dogs are always referred to as mixed-breed’. Table 1 shows some of the main demographic variables and the CCD scores regarding the breed types in our sample. The sample contained entries from *N* = 206 mixed-breed and *N* = 652 purebred dogs (*N* = 365 males and *N* = 493 females). From among the purebreds, *N* = 208 dogs belonged to the independent working breeds, *N* = 394 dogs were from cooperative breeds, and *N* = 50 dogs came from ‘toy’ breeds. We performed the functional breed-type assignments according to the description of the dogs’ original working tasks in the breed standards. In the case of toy breeds, the original function is providing companionship. We also considered a breed to be a ‘toy’ when the dogs from the breed are predominantly not being used for their original working task anymore, but for companionship only (e.g., Yorkshire Terrier, English Bulldog). The oldest dog in the sample was 19 years old, while the average age was 10.74 years. The average CCD score in the sample was 36.28 (minimum value = 17, maximum value = 74). In the case of *N* = 35 dogs the CCD score was 50 or above, which is the ‘dementia-threshold’ according to Salvin et al. (15). According to the owners, *N* = 545 dogs had regular sports/work activity. Many dogs have been involved in more than one type of sport. Most frequently mentioned sports were agility, obedience, IGP (Schutzhund), scent-work, herding, coursing. Remarkably, at the time of the questionnaire’s completion, *N* = 286 dogs were still actively doing sports/work. After excluding incomplete submissions, *N* = 858 entries (from which *N* = 77 human participants were male) were eligible for data analysis [*N* = 550 from Hungary and *N* = 308 from other countries, where the next most numerous entries (*N* = 225) came from the United States of America]. The known age distribution of participating dog owners was: (18–25 years) *N* = 47; (26–40 years) *N* = 224; (41–65 years) *N* = 431; (65+) *N* = 151. With regard to their highest education, *N* = 176 participants indicated high school, *N* = 633 indicated college.

**Table 1:** The breed types (first column) and the main demographic variables, including the CCD scores according to the questionnaire.

Breed type	Most frequent breed in sample	Sex ratio (male:female)	Average age ± SD (years)	Average CCD score (and range min/max)	Sports activity (yes/no)
Independent working dogs	Dachshund (*N* = 19)	92:116	10.6 ± 2.7	35.8 (17/66)	118/90
Cooperative working dogs	Labrador Retriever (*N* = 52)	163:231	10.5 ± 2.4	35.9 (17/70)	307/87
Toy breeds	Bichon Havanese (*N* = 8)	22:28	10.7 ± 2.8	37.3 (20/53)	16/34
Mixed breeds	N/A	88:118	11.4 ± 2.7	37.3 (19/74)	91/115

### 2.4 Statistical analysis

We performed all statistical tests with the IBM SPSS (Version 29) software. The level of significance was *α* = 0.05. The normality of the continuous variables was checked by the visual inspection of the Q–Q plots. We included to the initial models the biological relevant 2-way interactions, and then with backward model selection, we removed the non-significant interactions one by one. We report everywhere the final (simplest) models’ results.

With one-way ANOVA, we analyzed whether dogs’ age in the sample was associated with any of the fixed factors (breed type, sex, reproductive status, other dog in the household, status of the dog, end of sports career, walking frequency, and joint activity frequency).

The CCD-score of the dogs was analyzed with separate one-way ANOVAs, to detect potential associations with various sets of fixed factors. Breed type and its 2-way interactions with the other factors have been added to each initial model. We used the numerous fixed factors after sorting them into smaller groups to separate models, because we wanted to avoid the over-parametrization of the analyses. First, we analyzed whether CCD score was associated with breed type, breeding line, sex, reproductive status, housing and dog’s role. Next, we analyzed the associations between CCD score and breed type, age, height and weight of the dog. Third, we analyzed the associations between CCD score, breed type and the activity-related factors (other dog in the household, walking frequency, occurrence of sport/work activity, training level, joint activity frequency, end of sports career). Finally, in a separate analysis we investigated the CCD score’s association with a set of environmental and health-related factors (locality type, residence type, housing, age, body condition, injuries).

We also wanted to know whether the CCD score was associated with the owners’ reasons for having those dogs which were involved in this study. For this, we first ran a Principal Component Analysis (PCA) with each question about the reasons why the owners chose the dog. The PCA used the between-variable correlations with varimax rotation. We used the break point of the scree plot (42) for deciding the number of eventual PCA components. Items with loadings <0.25 were automatically disregarded. We applied the backward elimination approach, where we step-by-step excluded those parameters that had low loadings (<0.5), or which contributed to more than one component with similar absolute loading. [This approach is commonly used in PCA analysis, e.g., (43)] Cronbach’s alpha was calculated to assess the internal consistency of the final extracted factors and for testing the repeatability of the measurement (44). In behavioral studies, Cronbach’s alpha values above 0.6 are considered satisfactory [e.g., (45)]. The resulting components were used as dependent variables in the subsequent one-way ANOVAs, where CCD Score and dog’s age were added as fixed factors.

With the PCA, we found three reliable components (Table 2), which explained 62.164% of the total variance.

**Table 2:** The three components found with the Principal Component Analysis.

Questions/components	‘Ideal Dog’	‘Fancy Dog’	‘Breeding Dog’
Activity	0.779		
Health & longevity	0.758		0.304
Amicability	0.717		
Size	0.691		
Rarity		0.844	
Fashion		0.814	
Rescue			−0.787
Breeding			0.751
**Cronbach’s alpha**	**0.731**	**0.641**	**0.638**

In the first column we show the questionnaire items, with each of these the dog owners could indicate on a 1–5 Likert scale, how important the given characteristic was to them when they chose the dog. Numbers in the table are the factor loadings. In the bottom row we show the results of the reliability analysis (Cronbach’s alpha values).

*Component 1 (‘Ideal Dog’)*—According to the questionnaire items, in this case owners prioritized those features that are mainly important from the aspect of a healthy, pleasant companion animal.

*Component 2 (‘Fancy Dog’)*—This component comprises such features that emphasize the importance of special value of a dog (rarity and fashionableness).

*Component 3 (‘Breeding Dog’)*—The items of this component show an inverse relationship between two important factors in choosing a dog. Those who prioritized breeding their dog later, did not want rescue dogs, and those who preferred to adopt a dog from a rescue organization did not want to breed it later.

## 3 Results

At first, we analyzed whether dogs’ age had an association with some of the other fixed factors. Dogs’ age was significantly associated with the breed type, the end of sports career, the presence of other dog(s) in the family, the frequency of walks, and the frequency of joint activity with the dog (Table 3). The Tukey *post hoc* test did not show between-group differences in the case of the walk frequency.

**Table 3:** Results of the one-way ANOVA on the connection between dogs’ age and some of the fixed factors.

Fixed factor	*F*	Df, error	*p*
**Breed type**	**3.378**	**3, 834**	**0.018**
**Sports career’s end**	**38.214**	**2, 834**	**<0.001**
Sex	0.823	1, 834	0.365
Reproductive status	2.501	1, 834	0.114
**Other dogs in family**	**9.195**	**3, 834**	**<0.001**
Dog’s role	0.091	3, 834	0.965
**Walk frequency**	**3.333**	**4, 834**	**0.010**
**Joint activity frequency**	**2.898**	**4, 834**	**0.021**

Significant effects are highlighted with bold letters.

**Figure 1:**
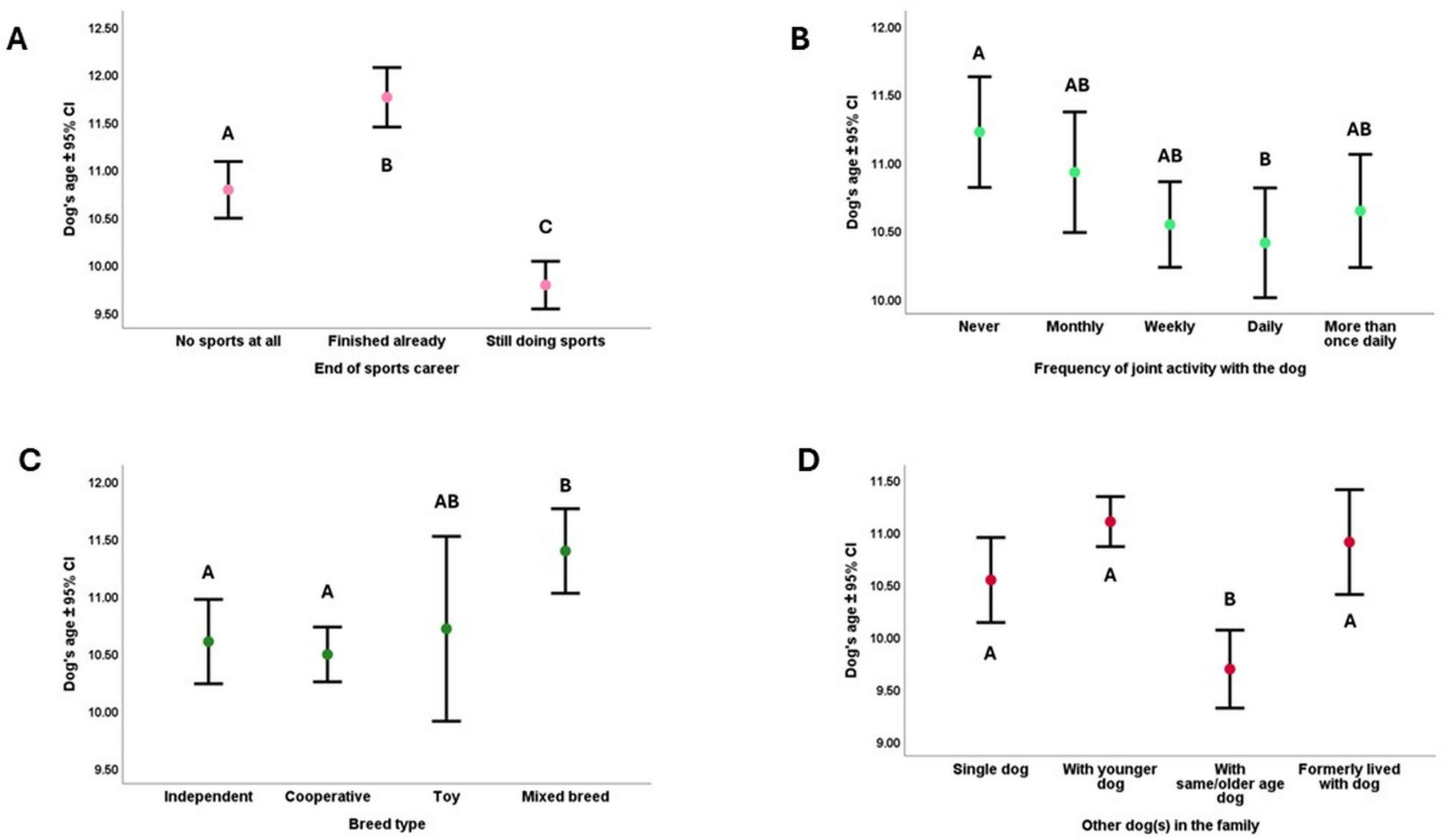
**(A–D)** The association between dogs’ age and **(A)** the end of their sports career; **(B)** the frequency of their joint activities with the owner; **(C)** their breed type; **(D)** the presence of other dogs in their homes. Different letters indicate significant between-group differences.

Based on their sports career’s end, each group significantly differed from the other. The oldest dogs have already finished their sports career; the youngest dogs were still doing sports. The age of those dogs who have never done any sport was in-between (Figure 1A). According to the *post hoc* tests, the oldest dogs in the sample did not do joint activities (e.g., fetching, tug-of-war, jogging, swimming) with the owners. However, the youngest dogs were having daily joint activity with the owner (Figure 1B). Mixed breed dogs were the oldest in our sample, the cooperative and independent working dog breeds the youngest, while toy breeds did not differ from the other three groups (Figure 1C). Finally, according to the Tukey *post hoc* tests, those dogs that lived together with the same age or older dogs (Figure 1D) were significantly the youngest dogs in our sample.

The first group of fixed factors that we checked for association with the CCD Score, consisted of Breed type and various demographic variables, such as Breeding line, Sex, Reproductive status, Housing and Dog’s role. We also analyzed the 2-way interactions of Breed type with all the other independent factors (Table 4).

**Table 4:** Results of the one-way ANOVA on the connection between dogs’ CCD score and the demographic factors of the dogs.

Fixed factor	*F*	Df, error	*p*
Breed type	0.733	3, 841	0.532
Breeding line	0.778	3, 841	0.506
Sex	0.006	1, 841	0.939
Reproductive status	0.128	1, 841	0.721
Housing	1.736	3, 841	0.158
**Dog’s role**	**2.756**	**3, 841**	**0.041**

Significant effects are highlighted with bold letters.

**Figure 2:**
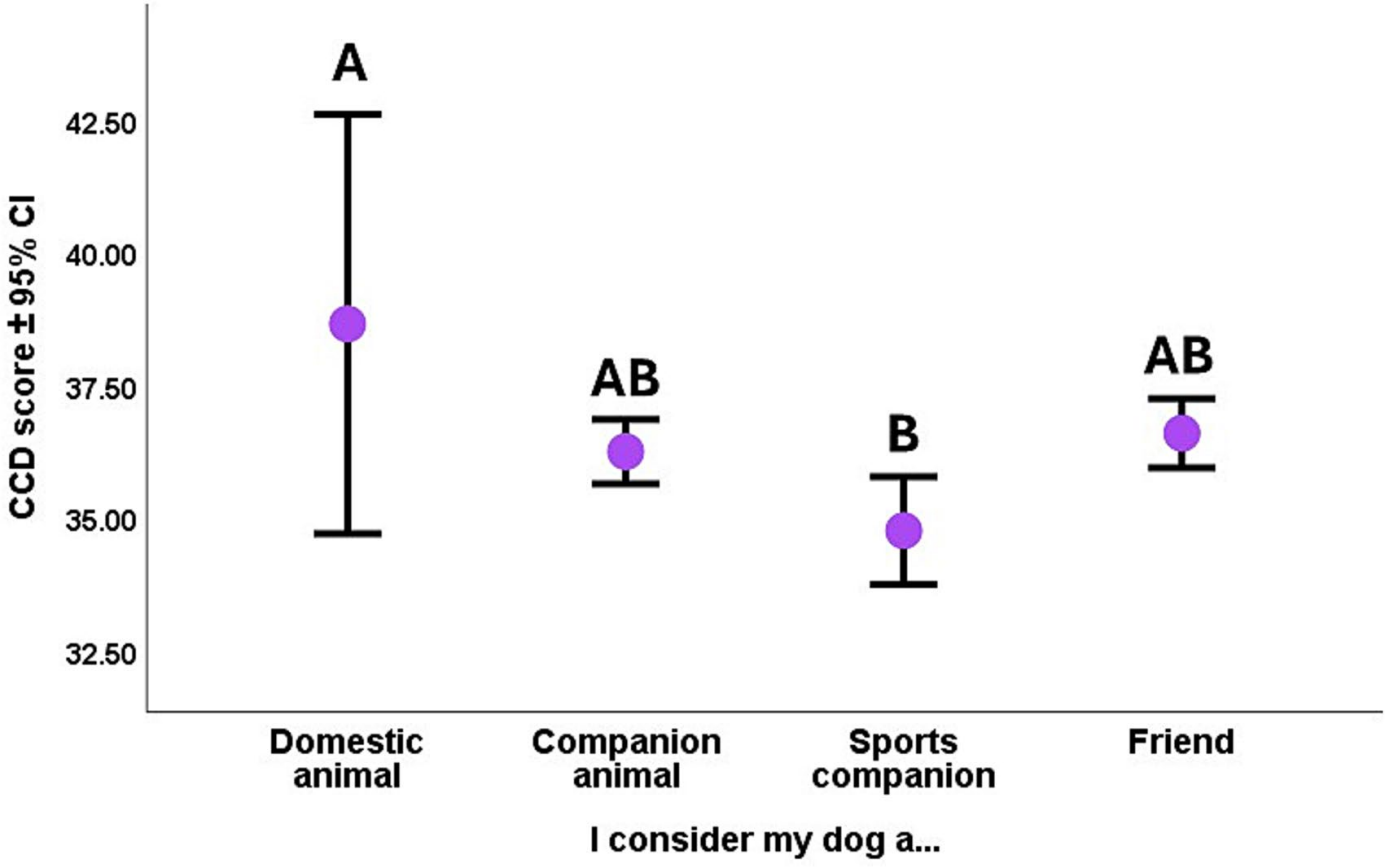
The association between dogs’ CCD score and their role according to the owner. Different letters indicate significant between-group differences.

Only the Dog’s role had a weak significant effect on the CCD score. (We clustered two categories together because of the low occurrence of subjects: ‘domestic animal’ and ‘breeding animal’). The Tukey post hoc test showed that regarding the Dog’s role, those dogs had significantly the highest CCD score who were considered ‘only’ as a domestic animal, and sports/working companions had the lowest CCD score (Figure 2).

In the next analysis, we checked if the CCD Score was in association with some of the dogs’ morphological features and age. Again, the initial model also contained the Breed’s two-way interactions with the other variables (Table 5). Only dogs’ age had significant effect on the CCD score.

**Table 5:** Results of the one-way ANOVA on the connection between dogs’ CCD score and the dogs’ breed type, age, weight and height.

Fixed factor	*F*	Df, error	*p*
Breed type	0.667	3, 578	0.572
Height	0.931	106, 578	0.671
Weight	0.813	122, 578	0.920
**Age**	**6.071**	**25, 578**	**<0.001**

Significant effects are highlighted with bold letters.

**Figure 3:**
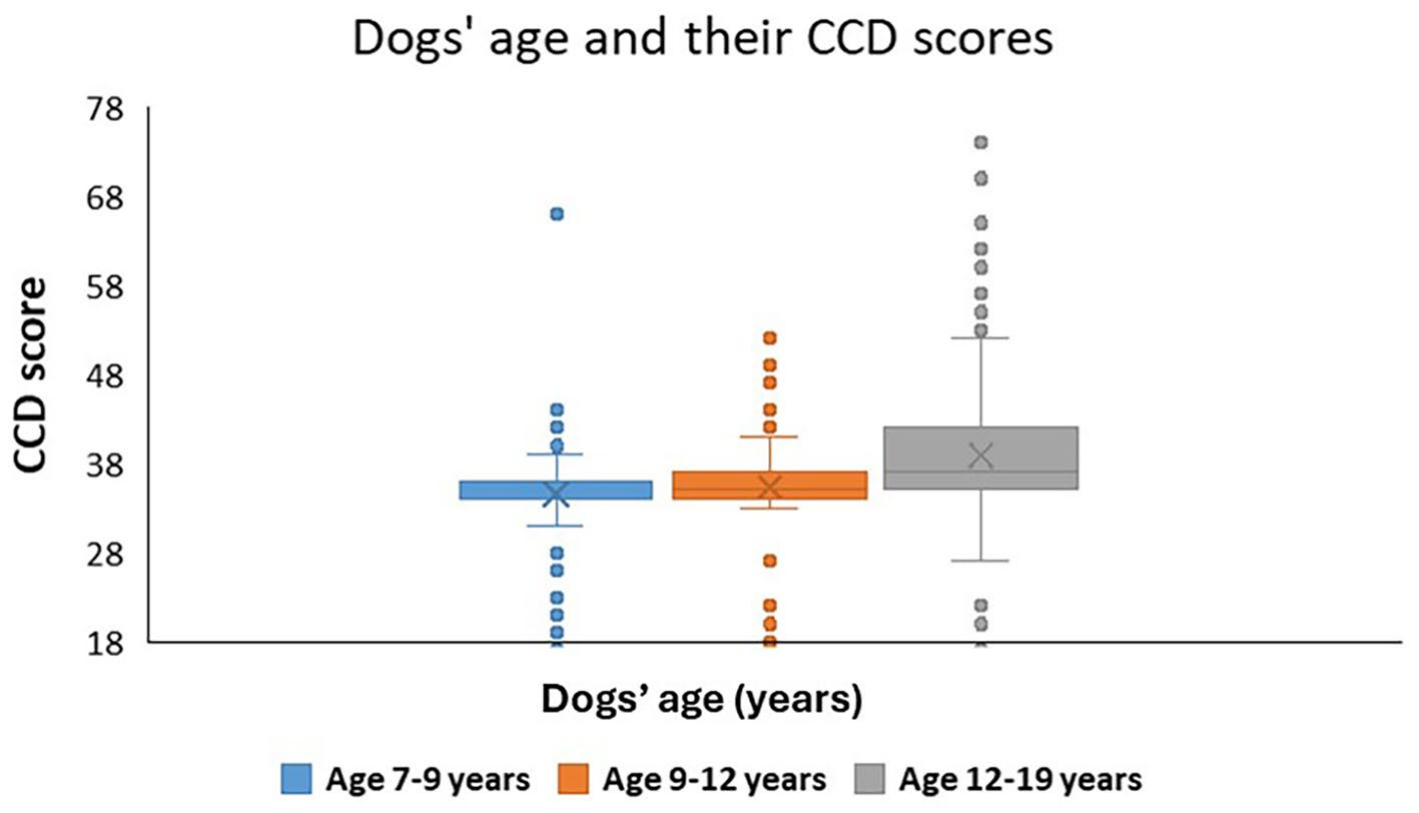
The association between dogs’ CCD score and their age. Age was used as continuous variable in the analysis, but for practical reasons for the illustration, we divided the sample into three age groups by using an equal number of dogs in each third.

CCD Scores were higher in the case of the older dogs in the sample (Figure 3).

Our next analysis targeted potential associations between the CCD Score and the various fixed factors of those conditions that could affect the activity level of the dog (other dog in the family, frequency of walks, occurrence of sport/work activity, training level, frequency of joint activities (with the owner), and the end of sports career) (Table 6).

**Table 6:** Results of the one-way ANOVA on the connection between dogs’ CCD score and the dogs’ breed type, other dog in the family, frequency of walks, occurrence of sport/work activity, training level, frequency of joint activities (with the owner), and the end of sports career.

Fixed factor	Df, error	*F*	*p*
Breed type	3, 820	1.107	0.345
*Other dog in family*	*3, 820*	*2.569*	*0.053*
Frequency of walks	4, 820	1.339	0.254
**Frequency of joint activity**	**4, 820**	**2.574**	**0.037**
Training level	5, 820	0.842	0.520
Occurrence of work	1, 820	0.541	0.462
**Sport career end**	**2, 820**	**13.359**	**<0.001**
Breed type * Walk freq.	*12, 820*	*1.660*	*0.071*

Significant effects are highlighted with bold letters; non-significant trends are highlighted with italics.

**Figure 4:**
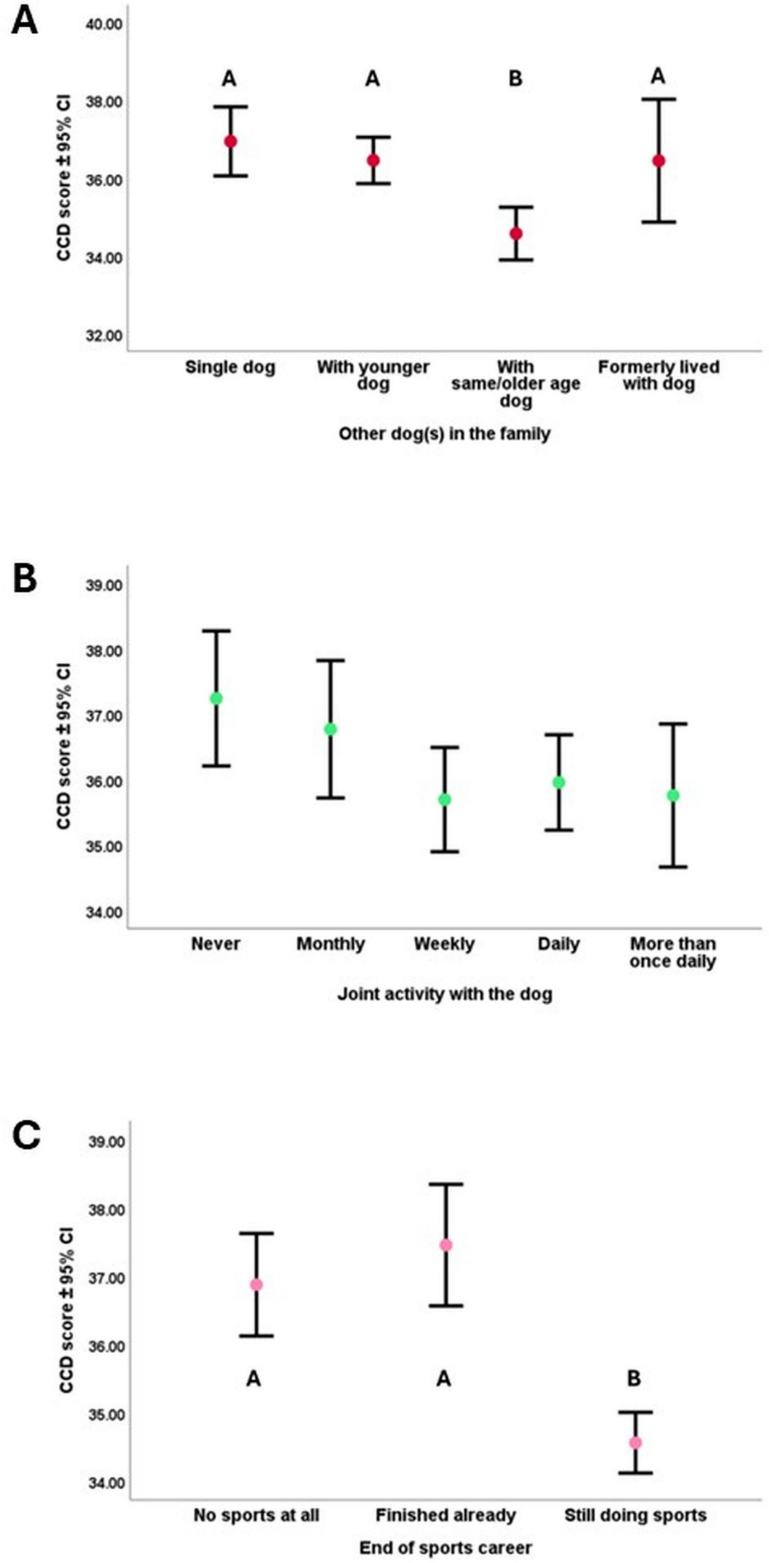
**(A–C)** The association between dogs’ CCD score and **(A)** the presence of other dog(s) in the same household; **(B)** the frequency of joint activities with the owner; **(C)** the end of their sports career. Different letters indicate significant between-group differences.

CCD score was in significant association with the frequency of joint activities and with the end of sports career. We also found a near-significant association with the presence of other dog(s) in the family, plus breed type with frequency of walks interaction, also had a trend-like effect on the CCD score. Those dogs had significantly lower CCD scores, who were accompanied by the same age or older dogs (Figure 4A).

In the case of joint activities with the owner, although we had a significant main effect, the *post hoc* test showed only trend-like differences between the various frequencies of joint activity. Accordingly, those dogs tended to have the highest CCD score with whom their owner did not do joint activities. Those dogs had lower CCD scores that did joint activities with the owner at least weekly (Figure 4B).

We found the strongest association of the CCD score with the dogs’ sports career end. Those dogs who were still doing sport/work activity had significantly lower CCD scores than those dogs who have never done sports or who have already ended their sports career (Figure 4C).

**Figure 5:**
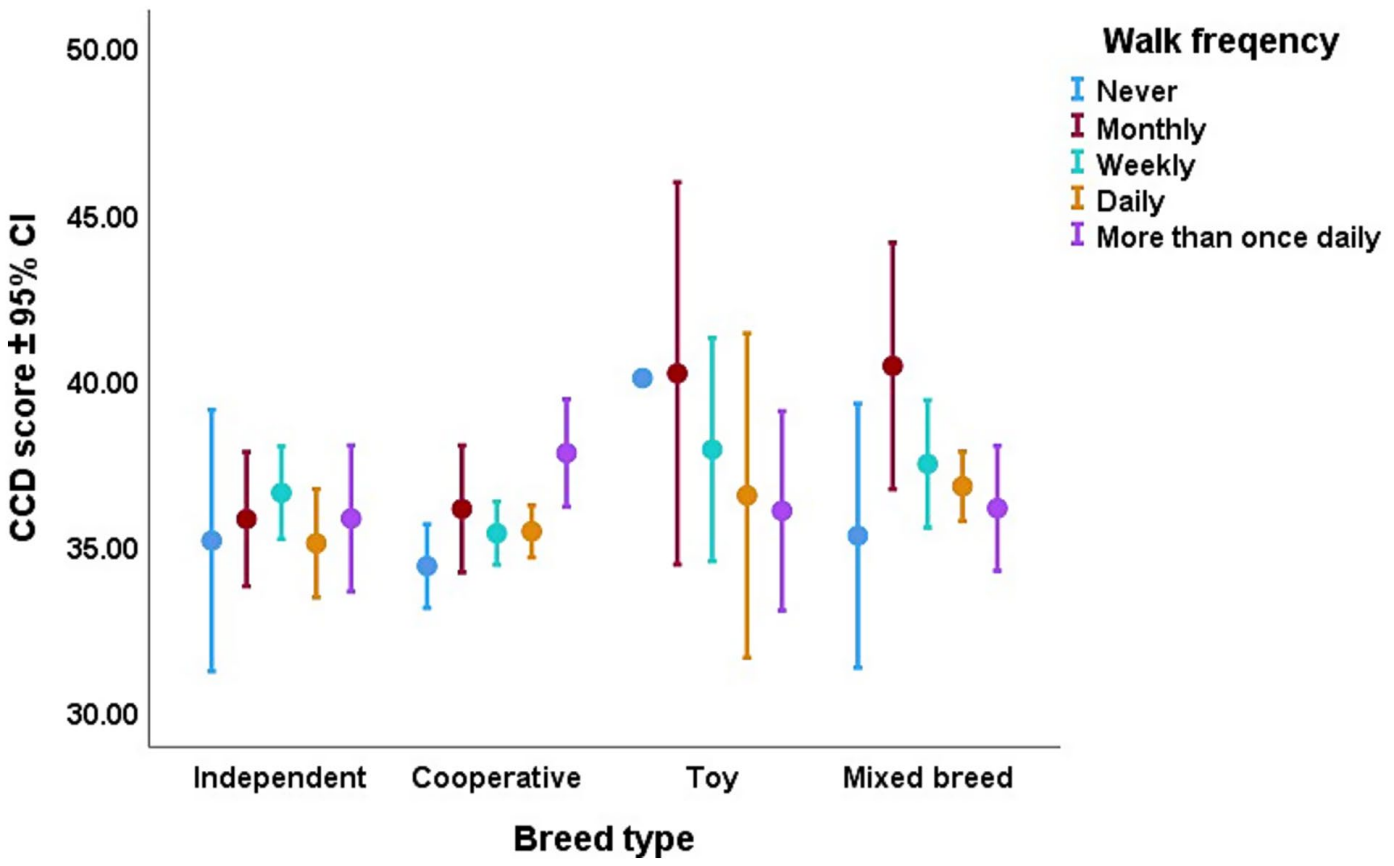
The trend-like effect of the interaction between breed type and frequency of walks on the dogs’ CCD score. More frequent walks had a more pronounced effect on CCD score in toy and mixed-breed dogs.

According to the non-significant trend effect on the interaction of breed type and frequency of walks, the more frequent walks could have a more positive effect (i.e., lower CCD score) in toy dogs and mixed breeds, than in the working dog breeds (Figure 5).

In the last group of fixed factors, we analyzed the associations of the CCD score with the various environmental factors (locality and residence type, housing), plus those factors that could affect the dog’s activity levels (Injury, Body condition and Age) (Table 7).

**Table 7:** Results of the one-way ANOVA on the connection between dogs’ CCD score and the dogs’ body condition, occurrence of injury, owner’s residence and locality where they live, and housing conditions.

Fixed factor	Df, error	*F*	*p*
*Breed type*	*3, 785*	*2.425*	*0.064*
Locality	4, 785	0.710	0.585
**Residence**	**3, 785**	**3.150**	**0.024**
Housing	3, 785	0.763	0.515
Body condition	4, 785	1.740	0.139
Injury	1, 785	1.354	0.245
Body_condition * age	**27, 785**	**2.972**	**<0.001**
**Age**	**25, 785**	**5.405**	**<0.001**

Breed type and dog’s age has been added to the analysis as well. Significant effects are highlighted with bold letters; non-significant trends are highlighted with italics.

**Figure 6:**
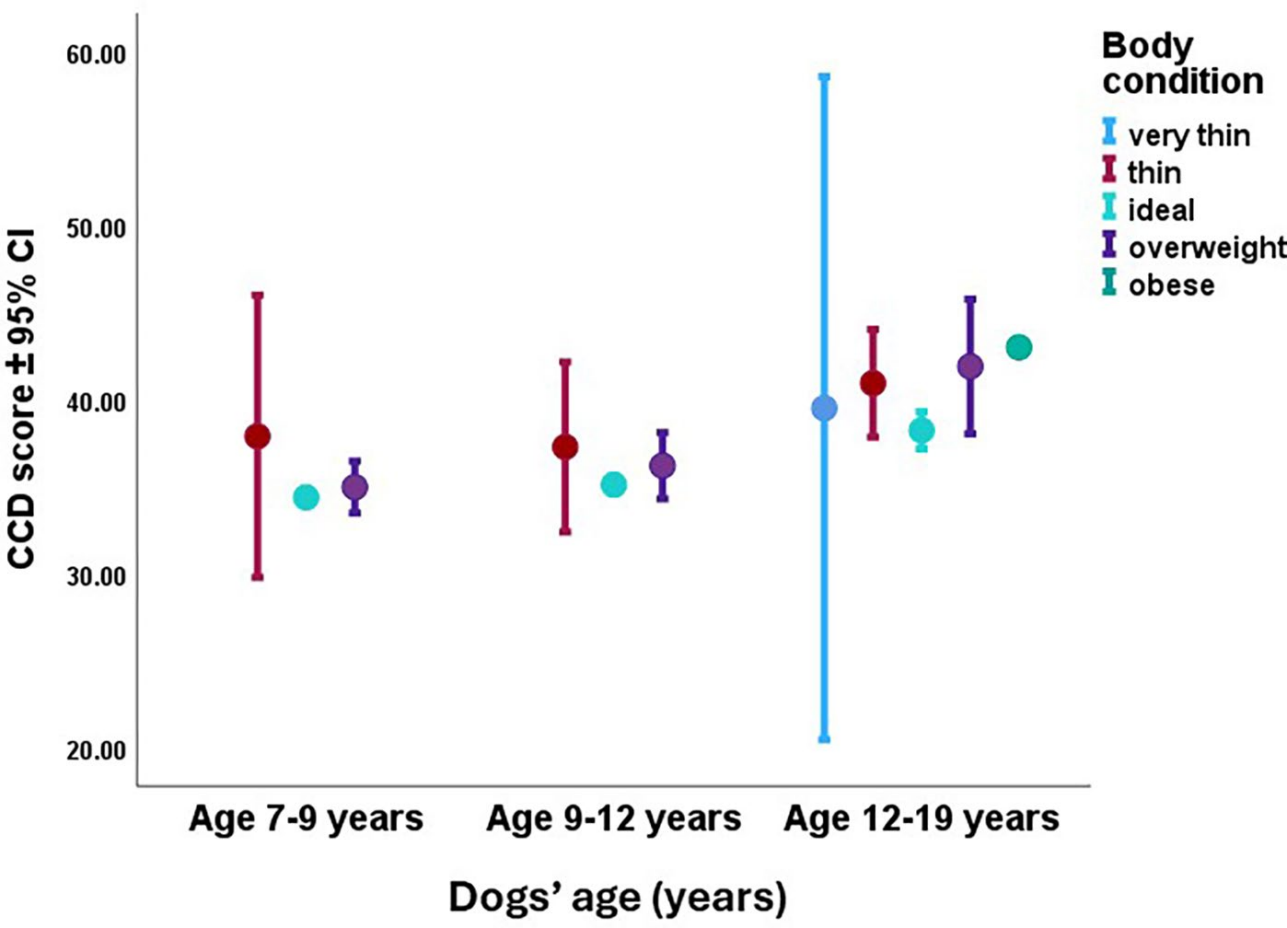
The effect of the significant interaction between age and body condition on the dogs’ CCD score. In the oldest third of the subjects, the more overweight the dogs were, the higher CCD scores they had. Age was used as a continuous variable in the analysis, but for practical reasons for the illustration, we divided the sample into three age groups by using an equal number of dogs in each third.

While Breed type had a trend-like association with the CCD score, we found a significant effect of residence, and a strong significant effect of the interaction between dogs’ age and body condition. In the case of residence, dogs who lived in a home with their own garden had lower CCD scores than those dogs who lived in an apartment without a garden, or in a home that had a common garden shared with other inhabitants. According to the interaction (Figure 6), body condition also had a clearer association with CCD score in the case of the oldest third of the subjects. Meanwhile in the younger cohorts, body condition had no association with the CCD score, the oldest dogs who were also overweight had higher CCD scores than if they were thin or ideal weight.

The analysis of the connection between CCD score of the dogs and the principal components regarding the owners’ initial choice considerations revealed a significant effect of the CCD score in the case of ‘Ideal Dog’ and ‘Breeding Dog’ components. Dogs’ age did not associate significantly with CCD score in these models. ‘Fancy Dog’ choice score did not associate significantly with the CCD score (Table 8).

**Table 8:** Results of the one-way ANOVA on the connection of dogs’ CCD score and age with the three principal components regarding the reasons why the owner chose the dog.

Principal component	Fixed factor	*F*	Df, error	*p*
‘Ideal Dog’	Age	1.141	25, 773	0.289
	**CCD score**	**1.404**	**46, 773**	**0.042**
‘Breeding Dog’	Age	0.785	25, 773	0.764
	**CCD score**	**1.684**	**46, 773**	**0.004**
‘Fancy Dog’	Age	1.302	25, 773	0.148
	CCD score	0.798	46, 773	0.830

Significant effects are highlighted with bold letters.

**Figure 7:**
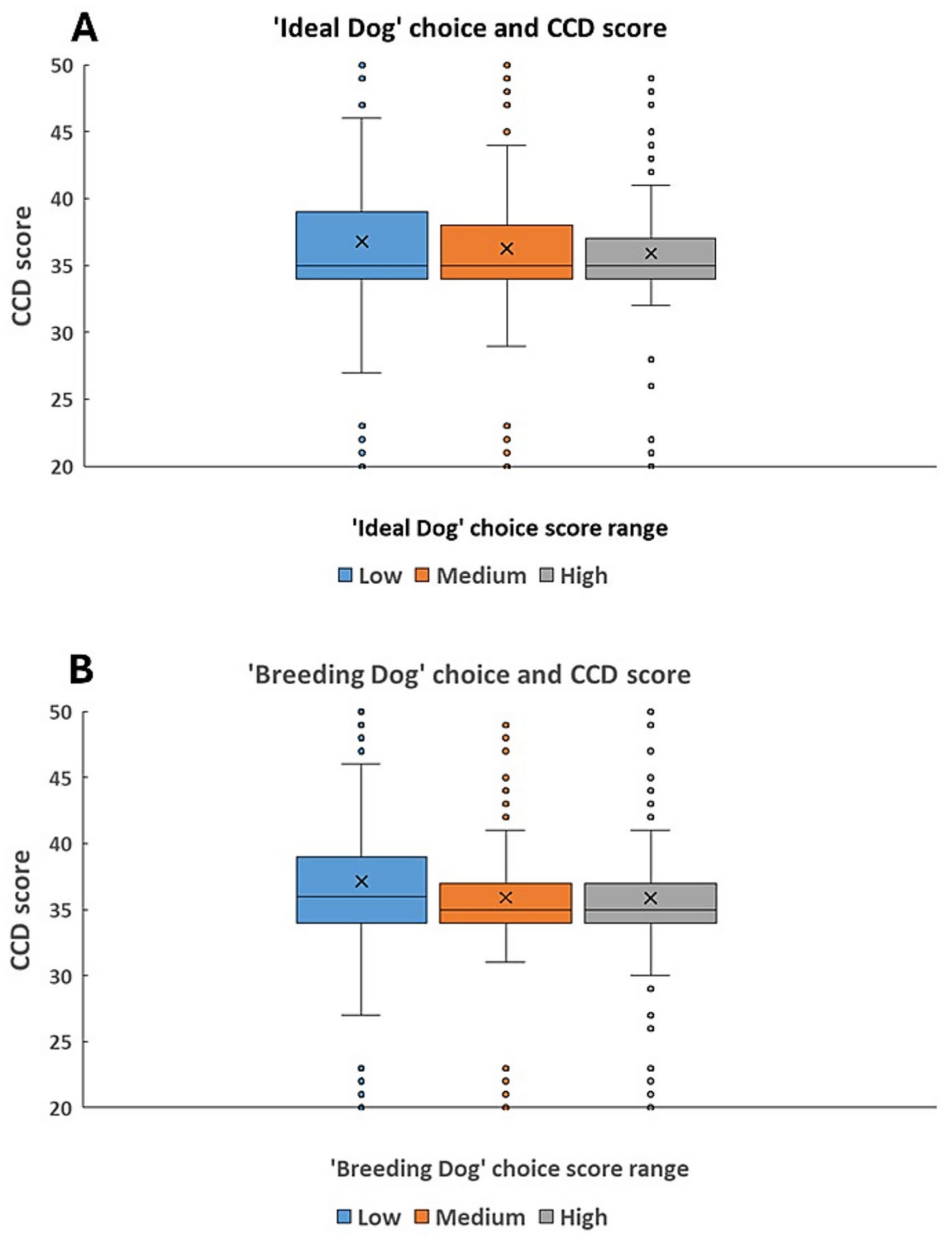
**(A,B)** The association between dogs’ CCD score and **(A)** the ‘Ideal Dog’ principal component; **(B)** ‘Breeding dog’ principal component. ‘Ideal Dog’ principal component showed how important the following traits were for the owners: dog’s size, amicability, activity level, and health/longevity. ‘Breeding dog’ principal component showed how important the following traits were for the owners: dogs should be good for breeding and dogs should not come from rescue. Both scores were used as a continuous variable in the analysis, but for practical reasons for the illustration, we divided the sample into three groups by using an equal number of dogs in each third of the score range.

In both cases of the ‘Ideal Dog’ (Figure 7A) and ‘Breeding Dog’ choice scores (Figure 7B), we found that the higher the priority owners put on these aspects, the dogs had lower CCD scores. These results were not affected by age.

## 4 Discussion

In our questionnaire we aimed to detect breed-type and activity-related differences in elderly dogs’ cognitive decline tendencies. In our sample of elderly dogs, the main measure of cognitive decline, CCD score ranged between 17 and 74. We should keep it in mind that in the whole sample, only 35 dogs had >50 CCD score, thus, the majority of the dogs in this study showed normal levels of age-related cognitive decline instead of accelerated process of dementia. Summarizing our main findings, the old-age cognitive decline of dogs has not been associated with functional breed types, however, the lifetime activity had a significant effect on Canine Cognitive Decline (CCD scores). Everyday joint activities with the owner, as well as lifetime sport activity (signified by still ongoing sports career in many of the old dogs versus already ceased or never existed sports engagement), resulted in lower CCD scores. Therefore, our study gave the first indication that participating in dog sports can postpone the development of cognitive decline. We also found that when dog owners had clearer goals and choices for their next dog (e.g., they sought information about the dog’s health, longevity, activity levels and size), these dogs had lower CCD scores, which indicates either the owners’ more conscientious efforts to provide the necessary cognitive stimulation to their dogs, and/or the effect of a more careful choice on the biological qualities of the acquired dog was also important.

In our sample, mixed breed dogs were the oldest, and dogs from the working breed types were the youngest, this result falls in line with studies that showed longer life expectancy was found for mixed breed dogs (46). Lifespan in dogs can be influenced by the higher genetic variability among mixed breeds who are less affected by inbreeding. It was found that the Genetic Illness Severity Index for Dogs (GISID) was in positive correlation with the level of inbreeding of dogs, and both indices were the highest among purebreds and lowest among mixed-breeds (‘mongrels’) based on a large (>30,000) dataset in the UK (47). The same study also found that mixed-breeds had longer lifespan than purebred or cross-bred dogs. On the other hand, toy breeds showed a wider age distribution in our sample. Most toy breeds belong to smaller sized dogs, which have a longer life expectancy than the larger working breeds do (48).

We found that older dogs had already ended their sport career, which could be that eventually, the owners decided to finish their vigorous exercise with them as well. However, it might also be that sports dogs were still in good condition at the beginning of their older age, thus they can stay healthy longer even after their retirement. This is also supported by the result that with younger dogs, owners did more joint activities daily, perhaps this shows that the oldest dogs were unable to do joint activities further. Moreover, we found that those dogs which had the highest CCD score, were the ones that did not do joint activities with their owner at all. The dogs that had lower CCD scores were those that did joint activities with their owner at least weekly. These associations need to be further investigated, as the causative relationship is possible in both directions. However, it is more likely that minimum once a week joint activity, may prevent or delay the formation of cognitive decline as previous studies have found that physical and cognitive training had a positive effect on old dogs’ cognitive abilities (32). Another, trend-like result of our study emphasized the importance of frequent walks. Although walks are not as physically stimulating as regular sports activity and training, frequent walks still had a more positive effect (i.e., lower CCD score) for toy dogs and mixed breeds than in the working dog breeds. A possible explanation for this could be that as working dog breeds presumably do sports more frequently, in their case, walks played a less important role than in the case of the mixed breed and toy dogs. For elderly toy and mixed breed dogs, no walks could basically mean a life without physical exercise, which, according to our results, can lead to higher CCD scores than in working breeds without frequent walks. As mixed breed dogs were the oldest in the sample (and the toy breeds were next in line), the benefits of frequent walks in their case are especially remarkable. This result falls in line with the findings of the large-scale Dog Aging Project, which showed that small sized dogs were, in general, less active than large-bodied dogs (49). Overall, similar to the findings in the case of humans (27, 28), long-term and regular physical activity might reduce the risk of dementia-like canine cognitive decline. It was even assumed that in dogs, behavioral enrichment may promote neurogenesis later in life (50).

Importantly, not only joint activities, but participating in sport/work also affected dogs’ cognitive decline. Dogs with current, regular and frequent (i.e., more than once a week) sport activity had lower CCD scores than those that either have never done sport before or have already finished their sports career. However, it is important to note that in our sample, dogs with ongoing sport activities were the youngest. On the other hand, while retired sports dogs were the oldest in the sample, the non-sporting dogs, who were otherwise younger, still had similarly high CCD scores compared to them. This result further highlights the potential positive effect of sports for delaying the development of old age cognitive decline. Moreover, the result that dogs who have never done sports were younger than retired sporting dogs, could be extrapolated to the life expectancy of dogs with or without lifetime sports engagement. Hence, it is plausible that sport dogs would outlive the non-sporting subjects. Therefore, our study gives the first potential evidence that lifetime sport activity has positive effect on aging, and it may also extend life expectancy in dogs. At the same time, we should not forget that in our questionnaire study the participating dogs’ physical condition has not been evaluated. Others found that old-age related musculo-skeletal pain can reduce the dogs’ willingness to move (51), leading to a vicious circle of lack of physical activity and declining cognitive functioning. The potential connection between regular physical activity and the onset of joint-originated painful conditions would need further investigation.

Another interesting result revealed that those dogs that were treated as domestic animals or breeding animals, had higher CCD scores than working companions, who had the lowest CCD scores. Previous studies also found that the owner’s management style and characteristics, are connected to the role they attribute to the dog, but they did not find association with canine behavioral problems reported by the owners (52). However, so far, our study is the first that has found a connection between the cognitive decline of dogs and the role attributed to them by their owners. We should also consider another interesting parallel between our results and the positive associations found between the stockpersons’ attitude towards livestock and the productivity of these animals. According to Hemsworth (53), attitude is a major antecedent of the stockperson’s actual behavior and handling style towards pigs and dairy cattle. Beliefs and opinion about the animal are part of the human attitude towards them, and we can assume that in our study dog owners who considered their dogs as being friends or family members, provided more beneficial social engagement to them than those owners who considered their dog as a domestic or breeding animal.

In our study the ‘Dog’s role’ did not show association with the age of dogs, thus, regular sport activity might indeed relate to the lower CCD scores. Similarly, we found that the higher priority owners put on the aspects of choosing an ‘Ideal’ or a ‘Breeding’ dog, the lower the dogs’ CCD scores were, which results were not confounded by the age of the dog. This result suggests that owners with clearer goals for choosing a dog most likely also provided the best, stimulating environment to their canine companions. This idea is supported by earlier results, where it was found that prospective owners who did more extensive research about the needs and qualities of their dog and best practices of dog keeping, they also knew more about basic welfare issues, realistic costs of dog ownership and possible solutions of handling behavioral problems (54). It is also possible that if the dog was well-chosen according to the principles of ‘Ideal Dog’ or ‘Breeding Dog’, it already had such qualities that somewhat prevented the development of old age CCD.

Younger dogs in our sample tended to live together with either same age or older dogs. Although a simple explanation arises, that those dogs must be the youngest, who have still older companions at home, this does not explain why the single-kept dogs were also significantly older in our sample. Moreover, this would only resolve the difference between the two groups where the subject dog lives with younger or older companions. Dogs with same age or older companions had the lowest CCD scores as well. In humans, loneliness and isolation was found to relate to heightened risk of dementia, where the neural biomechanisms in the background included brain volume alterations at specific areas as well as changes in cortisol secretion (55). Although companion dogs have at least access to their owners, the cognitively and physically stimulating effect of conspecifics cannot be ignored. Of course, as single-kept dogs were in general older than the dogs who had same age or older companion dogs, they might reach higher CCD scores simply due to their higher age, too. The effect of a conspecific companion seems to be beneficial against cognitive decline, at least at the beginning of the old age period in dogs. However, according to the results, in truly old dogs, the presence of younger companions does not seem to lower the CCD scores.

From among the environmental factors, type of residence showed a significant association with CCD scores: those dogs who had access to a private garden of their home, had lower CCD scores compared to the dogs living without a garden or with a shared garden with other inhabitants. This result may indicate that similarly to cats, dogs can also find outdoor activities positively stimulating (56), either alone or with their owner. When the residence has an adjacent private garden, this feature can also provide a convenient opportunity for the owner or family members to engage in joint playful activities with the dog, compared to other types of residence, where reaching suitable outdoor areas poses a more challenging task.

The older the dogs were, the higher CCD scores they had, which fits to the vast literature of age-related cognitive decline in dogs [e.g., (14)]. Furthermore, while in the younger cohorts, body condition had no association with the CCD score, the oldest dogs who were also overweight had higher CCD scores than if they were thin or ideal weight. This result can relate to the lack of physical exercise that can lead to obesity and accelerated cognitive decline at the same time. Obesity in old dogs can reduce the dogs’ willingness to move because of various reasons, such as lack of energy, orthopedic issues, and chronic systemic inflammation (57).

In humans, it was found that old-age dementia showed a positive association with mid-age overweight body condition (58). In dogs, according to various studies, body condition had no clear association with cognitive decline. While Katina et al. (24) found that dogs with uncontrolled access to food had higher CCD scores, others found that thin body condition can also be a risk factor in cognitive decline (59). Our study showed that among the aged dogs, body condition had a significant association with CCD score only in the oldest cohort, where more obese dogs had also higher CCD scores. As our questionnaire did not survey the body condition history of the subjects, it is unknown whether the very old obese dogs have been overweight for a long time, or they have only started to gain weight more recently. As obesity often coincides with lack of physical activity (60), we can assume that old, obese dogs, who may also spend most of the day inactive, the cognitive decline could accelerate.

### 4.1 Limitations

Convenience sampling-based questionnaire studies run via the internet inherently suffer from being non-representative, because potential participants can have different social media using habits and access to the internet. Furthermore, it is expected that the topic of the study immediately causes interest-based bias among the participants, for example in our case, it is likely that those who completed the questionnaire had an above-average preference towards issues with dog-welfare and sports activities. As an attempt to mitigate this problem, we tried to create a title for our questionnaire that was neutral and generic enough, but at the same time, highlighted that the research was about canine aging and whether the dog was involved or not in sports. Our study fully relied on the accuracy and honesty of the dog owners regarding their assessment of their dogs’ behavior, body condition and demographics. For example, earlier it was found that owners may refrain from reporting their dogs were obese or too thin in questionnaires (61). As our study was a cross-sectional endeavor, we cannot assess causality behind the found effects, for example, whether long-term sports engagement resulted in lower CCD-scores of the subjects, or the dogs that showed less cognitive decline could remain physically active longer. Furthermore, in our research we did not find sex related differences in the dogs’ CCD scores contrary to other studies (35), which found that female dogs had a higher chance for cognitive decline than males. However, as the subjects of our survey were predominantly spayed/neutered companion dogs, this could mask the potential sex-related results in cognitive decline. From this aspect, especially desexing of male dogs could have an effect on our results, as other studies found that intact males showed much slower cognitive decline than female and neutered male dogs (62).

## 5 Conclusion

Compared to convenience sampling of dog breeds, functional breed selection offers an ecologically valid framework for the investigation of breed-related behavioral differences in dogs (37). Contemporary behavioral and neuro-ethological methods opened up many opportunities to investigate the complex socio-cognitive capacities of dogs, both when they function properly and when they show problems (63). Our study was the first to investigate whether old age cognitive decline could be associated with the functional breed categories, lifetime sport activity, and joint activities with the owner. Our results showed that breed type (cooperative vs. independent working breeds) did not directly influence the severity of cognitive decline, however, we found several indications that both leisure and organized sport activity may prevent the onset of canine dementia. This notion gained support from various directions, for example when owners considered their dogs as sport companions, these animals had lower CCD scores than the dogs who were considered to be more like livestock, or merely ‘domesticated animals’ or ‘breeding animals’. Remarkably, dogs who were otherwise not selected for working tasks (toy breeds and mixed breeds) showed a stronger effect of joint activity with the owner as a preventive factor against high CCD scores. This indicates that working breeds, regardless of their cooperative or independent predisposition, could mentally benefit from even modest amounts of physical activity with the owner. However, toy breeds and mixed breed dogs, who may lack the strong drive for work-related tasks, may require more conscientious efforts from their owners to combat CCD. Potential advice for professionals and dog owners could be that working dog breeds might have an advantage to stave off old age cognitive decline as they have a stronger predisposition for physical activities. Joint activities with the owner may lack the rigor and physical intensity that is required for sports; however, their mental stimulative effect cannot be ignored. Toy breeds and mixed breed dogs, which have a relatively good chance of living longer lives, may be able to stay mentally fresh through joint activity with the owner, as this appears to have an especially promising potential. In the future, the findings of this questionnaire could provide relevant and testable hypotheses for longitudinal investigations of the various dog breed types exposed to specific activity/exercise regimes. In this case, signs of old age cognitive decline could be objectively detected with behavioral test batteries and by using molecular biomarkers such as the plasma neurofilament light chain (pNfL) concentration (64).

## Data Availability

The original contributions presented in the study are included in the article/Supplementary material, further inquiries can be directed to the corresponding author.

## References

[1] López-Otín C Blasco MA Partridge L Serrano M Kroemer G. The hallmarks of aging. Cell. (2013) 153:1194–217. doi: 10.1016/j.cell.2013.05.039, 23746838 PMC3836174

[2] Finch CE. Evolution of the human lifespan and diseases of aging: roles of infection, inflammation, and nutrition. Proc Natl Acad Sci USA. (2010) 107:1718–24. doi: 10.1073/pnas.0909606106, 19966301 PMC2868286

[3] Kumar A Singh A Ekavali. A review on Alzheimer’s disease pathophysiology and its management: an update. Pharmacol Rep. (2015) 67:195–203. doi: 10.1016/j.pharep.2014.09.004 25712639

[4] Morand-Ferron J Cole EF Quinn JL. Studying the evolutionary ecology of cognition in the wild: a review of practical and conceptual challenges. Biol Rev. (2016) 91:367–89. doi: 10.1111/brv.12174, 25631282

[5] Kutwad S Khankal S Prabhu A. Ageing in animals: Alzheimer’s like disease. Next Res. (2025) 2:101076. doi: 10.1016/j.nexres.2025.101076

[6] Townsend AK Williams KEG Nannas NJ. Inbreeding and cognitive impairment in animals. Behav Ecol. (2024) 36:arae101. doi: 10.1093/beheco/arae101

[7] Lemaître J-F Gaillard J-M Lackey LB Clauss M Müller DWH. Comparing free-ranging and captive populations reveals intra-specific variation in aging rates in large herbivores. Exp Gerontol. (2013) 48:162–7. doi: 10.1016/j.exger.2012.12.004, 23261518

[8] Brando S Chapman S Surgeon V. Optimal Wellbeing of Ageing Wild Animals in Human Care. Cham.: Springer (2023).

[9] Blanchard T Eppe J Mugnier A Delfour F Meynadier A. Enhancing cognitive functions in aged dogs and cats: a systematic review of enriched diets and nutraceuticals. Geroscience. (2025) 47:2925–47. doi: 10.1007/s11357-025-01521-z, 39827310 PMC12181554

[10] Pan Y. Nutrients, cognitive function, and brain aging: what we have learned from dogs. Med Sci (Basel). (2021) 9:72. doi: 10.3390/medsci9040072, 34842769 PMC8628994

[11] Dewey CW Davies ES Xie H Wakshlag JJ. Canine cognitive dysfunction: pathophysiology, diagnosis, and treatment. Vet Clin North Am Small Anim Pract. (2019) 49:477–99. doi: 10.1016/j.cvsm.2019.01.013, 30846383

[12] Chapagain D Range F Huber L Virányi Z. Cognitive aging in dogs. Gerontology. (2018) 64:165–71. doi: 10.1159/000481621, 29065419 PMC5841136

[13] Albert MS. Changes in cognition. Neurobiol Aging. (2011) 32:S58. doi: 10.1016/j.neurobiolaging.2011.09.010, 22078174 PMC3929949

[14] Bellows J Colitz CMH Daristotle L Ingram DK Lepine A Marks SL . Defining healthy aging in older dogs and differentiating healthy aging from disease. J Am Vet Med Assoc. (2015) 246:77–89. doi: 10.2460/javma.246.1.77, 25517329

[15] Salvin HE McGreevy PD Sachdev PS Valenzuela MJ. The canine cognitive dysfunction rating scale (CCDR): a data-driven and ecologically relevant assessment tool. Vet J. (2011) 188:331–6. doi: 10.1016/j.tvjl.2010.05.014, 20542455

[16] Amaducci L Lippi A. Risk factors for Alzheimer’s disease. Int J Geriatr Psychiatry. (1992) 7:383–8. doi: 10.1002/gps.930070602, 1414264

[17] Brookmeyer RS Gray SC Kawas C. Projections of Alzheimer’s disease in the United States and the public health impact of delaying disease onset. Am J Public Health. (1998) 88:1337–42. doi: 10.2105/AJPH.88.9.1337, 9736873 PMC1509089

[18] Azkona G Chacón G García-Belenguer S Rosado B León M Palación J. Prevalence and risk factors of behavioural changes associated with age-related cognitive impairment in geriatric dogs. J Small Anim Pract. (2009) 50:87–91. doi: 10.1111/j.1748-5827.2008.00718.x 19200264

[19] Pike CJ Carroll JC Rosario ER Barron AM. Protective actions of sex steroid hormones in Alzheimer’s disease. Front Neuroendocrinol. (2009) 30:239–58. doi: 10.1016/j.yfrne.2009.04.015, 19427328 PMC2728624

[20] Taupin P. A dual activity of ROS and oxidative stress on adult neurogenesis and Alzheimers disease. Cent Nerv Syst Agents Med Chem. (2010) 10:16–21. doi: 10.2174/187152410790780172 20236039

[21] Head E Rofina J Zicker S. Oxidative stress, aging, and central vervous system disease in the canine model of human brain aging. Vet Clin North Am Small Anim Pract. (2008) 38:167–78. doi: 10.1016/j.cvsm.2007.10.002, 18249248 PMC2390776

[22] Selhub J Bagley LC Miller J Rosenberg IH. B vitamins, homocysteine, and neurocognitive function in the elderly. Am J Clin Nutr. (2000) 71:614S–20S. doi: 10.1093/ajcn/71.2.614s, 10681269

[23] Cole GM Ma Q-L Frautschy SA. Omega-3 fatty acids and dementia. Prostaglandins Leukot Essent Fat Acids. (2009) 81:213–21. doi: 10.1016/j.plefa.2009.05.015, 19523795 PMC4019002

[24] Katina S Farbakova J Madari A Novak M Zilka N. Risk factors for canine cognitive dysfunction syndrome in Slovakia. Acta Vet Scand. (2015) 58:17. doi: 10.1186/s13028-016-0196-5, 26927954 PMC4772312

[25] Rohlf VI Toukhsati S Coleman GJ Bennett PC. Dog obesity: can dog caregivers’ (owners’) feeding and exercise intentions and behaviors be predicted from attitudes? J Appl Anim Welf Sci. (2010) 13:213–36. doi: 10.1080/10888705.2010.483871, 20563903

[26] Rohlf VI Bennett PC Toukhsati S Coleman G. Beliefs underlying dog owners’ health care behaviors: results from a large, self-selected, internet sample. Anthrozoös. (2012) 25:171–85. doi: 10.2752/175303712X13316289505341

[27] López-Ortiz S Lista S Valenzuela PL Pinto-Fraga J Carmona R Caraci F . Effects of physical activity and exercise interventions on Alzheimer’s disease: an umbrella review of existing meta-analyses. J Neurol. (2023) 270:711–25. doi: 10.1007/s00415-022-11454-8, 36342524

[28] Iso-Markku P Kujala UM Knittle K Polet J Vuoksimaa E Waller K. Physical activity as a protective factor for dementia and Alzheimer’s disease: systematic review, meta-analysis and quality assessment of cohort and case–control studies. Br J Sports Med. (2022) 56:701–9. doi: 10.1136/bjsports-2021-104981, 35301183 PMC9163715

[29] Groot C Hooghiemstra AM Raijmakers PGHM Van Berckel BNM Scheltens P Scherder EJA . The effect of physical activity on cognitive function in patients with dementia: a meta-analysis of randomized control trials. Ageing Res Rev. (2016) 25:13–23. doi: 10.1016/j.arr.2015.11.005, 26607411

[30] Head E. Brain aging in dogs: parallels with human brain aging and Alzheimer’s disease. Vet Ther. (2001) 2:247–60. 19746668

[31] Bray EE Raichlen DA Forsyth KK Promislow DEL Alexander GE MacLean EL . Associations between physical activity and cognitive dysfunction in older companion dogs: results from the dog aging project. Geroscience. (2023) 45:645–61. doi: 10.1007/s11357-022-00655-8, 36129565 PMC9886770

[32] Bognár Z Szabó D Turcsán B Kubinyi E. The behavioural effect of short-term cognitive and physical intervention therapies in old dogs. Geroscience. (2024) 46:5409–29. doi: 10.1007/s11357-024-01122-2, 38568435 PMC11493909

[33] Le Brech S Amat M Temple D Manteca X. Evaluation of two practical tools to assess cognitive impairment in aged dogs. Animals. (2022) 12:3538. doi: 10.3390/ani12243538, 36552458 PMC9774186

[34] McKenzie BA Chen FL. Assessment and management of declining physical function in aging dogs. Top Companion Anim Med. (2022) 51:100732. doi: 10.1016/j.tcam.2022.100732, 36273752

[35] Salvin HE McGreevy PD Sachdev PS Valenzuela MJ. Under diagnosis of canine cognitive dysfunction: a cross-sectional survey of older companion dogs. Vet J. (2010) 184:277–81. doi: 10.1016/j.tvjl.2009.11.007 20005753

[36] Kraus C Pavard S Promislow DEL. The size-life span trade-off decomposed: why large dogs die young. Am Nat. (2013) 181:492–505. doi: 10.1086/669665 23535614

[37] Pongrácz P Dobos P. Behavioural differences and similarities between dog breeds: proposing an ecologically valid approach for canine behavioural research. Biol Rev. (2025) 100:68–84. doi: 10.1111/brv.13128, 39101379 PMC11718627

[38] Pongrácz P Lugosi CA. Cooperative but dependent–functional breed selection in dogs influences human-directed gazing in a difficult object-manipulation task. Animals. (2024) 14:2348. doi: 10.3390/ani14162348, 39199881 PMC11350734

[39] Gácsi M McGreevy P Kara E Miklósi Á. Effects of selection for cooperation and attention in dogs. Behav Brain Funct. (2009) 5:1–8. doi: 10.1186/1744-9081-5-31, 19630939 PMC2731781

[40] Lugosi CA Udvarhelyi-Tóth KM Dobos P Pongrácz P. Independent, but still observant – dog breeds selected for functional independence learn better from a conspecific demonstrator than cooperative breeds in detour task. BMC Evol Biol. (2024) 22:245. doi: 10.1186/s12915-024-02046-1, 39444014 PMC11515571

[41] Dobos P Lugosi CA Pongrácz P. Do ostensive verbal signals have a unique importance when communicating with dogs? Evol Hum Sci. (2025) 7:e44. doi: 10.1017/ehs.2025.10031, 41445973 PMC12722044

[42] Cattell RB. The scree test for the number of factors. Multivar Behav Res. (1966) 1:245–76. doi: 10.1207/s15327906mbr0102_10, 26828106

[43] Cadima JFCL Jolliffe IT. Variable selection and the interpretation of principal subspaces. J Agric Biol Environ Stat. (2001) 6:62–79. doi: 10.1198/108571101300325256

[44] DeVellis RF Thorpe CT. Scale Development: Theory and Applications. Thousand Oaks: Sage publication (2021).

[45] Yordy J Kraus C Hayward JJ White ME Shannon LM Creevy KE . Body size, inbreeding, and lifespan in domestic dogs. Conserv Genet. (2020) 21:137–48. doi: 10.1007/s10592-019-01240-x, 32607099 PMC7326369

[46] Mata F Mata A. Investigating the relationship between inbreeding and life expectancy in dogs: mongrels live longer than pure breeds. PeerJ. (2023) 11:e15718. doi: 10.7717/peerj.15718, 37483958 PMC10362839

[47] Greer KA Canterberry SC Murphy KE. Statistical analysis regarding the effects of height and weight on life span of the domestic dog. Res Vet Sci. (2007) 82:208–14. doi: 10.1016/j.rvsc.2006.06.005, 16919689

[48] Lee H Collins D Creevy KE Promislow DEL Dog Aging Project Consortium Akey JM . Age and physical activity levels in companion dogs: results from the dog aging project. J Gerontol A Biol Sci Med Sci. (2022) 77:1986–93. doi: 10.1093/gerona/glac099, 35486978 PMC9536450

[49] Milgram NW Siwak-Tapp CT Araujo J Head E. Neuroprotective effects of cognitive enrichment. Ageing Res Rev. (2006) 5:354–69. doi: 10.1016/j.arr.2006.04.004, 16949888

[50] Mondino A Khan M Case B Giovagnoli S Thomson A Lascelles BDX . Activity patterns are associated with fractional lifespan, memory, and gait speed in aged dogs. Sci Rep. (2023) 13:2588. doi: 10.1038/s41598-023-29181-z, 36788306 PMC9929073

[51] Gillet L Simon B Kubinyi E. The role of dogs is associated with owner management practices and characteristics, but not with perceived canine behaviour problems. Sci Rep. (2024) 14:27548. doi: 10.1038/s41598-024-77400-y, 39532970 PMC11557872

[52] Hemsworth PH. Human–animal interactions in livestock production. Appl Anim Behav Sci. (2003) 81:185–98. doi: 10.1016/S0168-1591(02)00280-0

[53] Philpotts I Blackwell EJ Dillon J Tipton E Rooney NJ. What do we know about dog owners? Exploring associations between pre-purchase behaviours, knowledge and understanding, ownership practices, and dog welfare. Animals. (2024) 14:396. doi: 10.3390/ani14030396, 38338039 PMC10854595

[54] Guarnera J Yuen E Macpherson H. The impact of loneliness and social isolation on cognitive aging: a narrative review. J Alzheimers Dis Rep. (2023) 7:699–714. doi: 10.3233/ADR-230011, 37483321 PMC10357115

[55] Desforges E. Challenges and solutions surrounding environmental enrichment for dogs and cats in a scientific environment. Animals. (2021) 11:2980. doi: 10.3390/ani11102980, 34679999 PMC8532686

[56] Marchi PH Vendramini TH Perini MP Zafalon RV Amaral AR Ochamotto VA . Obesity, inflammation, and cancer in dogs: review and perspectives. Front Vet Sci. (2022) 9:1004122. doi: 10.3389/fvets.2022.1004122, 36262532 PMC9573962

[57] Kivimäki M Luukkonen R Batty GD Ferrie JE Pentti J Nyberg ST . Body mass index and risk of dementia: analysis of individual-level data from 1.3 million individuals. Alzheimers Dement. (2018) 14:601–9. doi: 10.1016/j.jalz.2017.09.016, 29169013 PMC5948099

[58] MacQuiddy B Moreno JA Kusick B McGrath S. Assessment of risk factors in dogs with presumptive advanced canine cognitive dysfunction. Front Vet Sci. (2022) 9:958488. doi: 10.3389/fvets.2022.958488, 36330158 PMC9622924

[59] Morrison R Penpraze V Beber A Reilly JJ Yam PS. Associations between obesity and physical activity in dogs: a preliminary investigation. J Small Anim Pract. (2013) 54:570–4. doi: 10.1111/jsap.12142, 24117778

[60] Eastland-Jones RC German AJ Holden SL Biourge V Pickavance LC. Owner misperception of canine body condition persists despite use of a body condition score chart. J Nutr Sci. (2014) 3:e45. doi: 10.1017/jns.2014.25, 26101613 PMC4473163

[61] Urfer SR Kaeberlein M. Desexing dogs: a review of the current literature. Animals. (2019) 9:1086. doi: 10.3390/ani9121086, 31817504 PMC6940997

[62] Berns G. Decoding the canine mind. Cerebrum. (2020) 2020:cer-04. doi: 10.3390/ani9121086 32395197 PMC7192336

[63] Fefer G Panek WK Khan MZ Singer M Westermeyer HD Mowat FM . Use of cognitive testing, questionnaires, and plasma biomarkers to quantify cognitive impairment in an aging pet dog population. J Alzheimer’s Dis. (2022) 87:1367–78. doi: 10.3233/JAD-215562, 35431246 PMC9177825

[64] Taber KS. The use of Cronbach’s alpha when developing and reporting research instruments in science education. Res Sci Educ. (2018) 48:1273–96. doi: 10.1007/s11165-016-9602-2

