# Supplementary material for: Hard-working or hardly working dogs stay young longer? Lifetime sports engagement, joint activity with the owner and breed type are associated with the severity of canine cognitive decline
Source: Front Vet Sci. 2026 Apr 28;13:1833531. doi: 10.3389/fvets.2026.1833531 (PMC13161145; doi:10.3389/fvets.2026.1833531)
Supplement: Supplementary file 2 [file Data_Sheet_1.pdf]

Hard-working or hardly working dogs stay younger longer?

Dear Participants!

We are happy to invite you to contribute to our research project, supported by the Morris Animal Foundation (USA) and the Department of Ethology at Eötvös Loránd University, Budapest, Hungary. Please, complete this questionnaire only if your dog is at least 7 years old. We have no other requirements, the dog can be purebred or mixed breed as well, your answers will be valuable in either case!

This research project is about the effects of regular work/sport-related activities on the cognitive aging of dogs. We would like to know whether the signs of old-age changes in your dog's behavior and thinking are affected by the dog's lifetime joint activity with you. Currently it is not known whether dogs who spent their life with regular sport/work-related activities, or dogs who just lived with their owner as a cheerful companion would show the signs of aging sooner and more intensely. By joining our research and completing the questionnaire, you and your dog can help us to find out the answer!

If you have more than one old dog, we would be happy if you could separately complete our questionnaire for each of them.

Completing this questionnaire may take 15 minutes. All collected data are handled confidentially. The questionnaire is anonymous, and the data are stored in a separate, password protected database which can only be accessed by the researchers involved in the study. The answers are not accessible by any third party without your permission and are only used for research purposes. After we have processed and analyzed the data, the results will be published in the form of scientific presentations, papers, or educational materials.

Consent statement:

I have read the above information. By participating in this research, I consent to my anonymized data being shared and treated as part of a larger database. I am aware that I may discontinue participating in this research at any time.

- o Yes

Owner demography

Age*:___________

Gender*

- o Woman
- o Man
- o Other

Highest education *

Mark only one option

- Primary school
- Secondary/high school
- University/College
- Other

Country you live in*______________

Type of living place*

Mark only one option

- Downtown
- Suburb
- Village
- Farmhouse
- Other

Type of residence*

Mark only one option

- Flat/apartment (without garden)
- Flat with shared garden
- House with garden
- Other

Dog demography

Breed*______________

Is it known, whether the dog is from a specified breeding program (show/working/not specified)? *

Mark only one option

- Show
- Working/sport
- Not specified
- Other/ I do not know

Does the dog have a pedigree? *

- Yes
- No

How tall is your dog, measured at the withers? * _______________ (cm)

Approximately how heavy is your dog? *_________________ (kg)

How old is your dog?*________________(years)

Sex*

- Male
- Female

Reproductive status*

- Intact
- Spayed/Neutered

Age when it was spayed/neutered (if your dog is intact, please skip this question):

____

Did the dog have puppies/sire a litter? *

Mark only one option

- No, never
- Yes, once or twice
- Yes, three or more times

Where was the dog obtained from? *

Mark only one option

- It was born at my house/residence
- I got it from a breeder
- I got it from a friend or family member
- I got it from a breed-specific rescue organization

- I got it from a shelter
- I found it (rescued from the street, forest etc.)

Housing/keeping conditions*

Mark only one option

- Only indoor
- Only outdoor
- Indoor and outdoor
- Kennel

Is this the only dog of the family or does/did it live with another dog for a longer period of time? *

Mark only one option

- It was only dog in his/her entire life
- It has a younger/same age cohabitant dog
- It has an older/same age cohabitant dog
- It had a cohabitant dog for a longer period earlier

Is this your first dog, or did you have other dogs before? *

Mark only one option

- This is my first dog
- I had one other dog before
- I had two or three other dogs before
- I had four or more other dogs before

Did or does your dog live together with another companion animal at home (e.g. cat, ferret, rabbit etc.) for a longer period? *

- Yes
- No

Activity with the dog

What is this dog for you? Please mark the single most important option*

Mark only one option

- Domestic animal

- Breeding animal
- Companion animal
- Sport/work companion
- Friend/family member/child

How important were the following factors when you chose this particular dog? *

	1. Not important	2.	3.	4.	5. Very important
Look	O	O	O	O	O
Longevity	O	O	O	O	O
Breed	O	O	O	O	O
Size	O	O	O	O	O
Function (original task of the breed)	O	O	O	O	O
Fashion/trend/popularity	O	O	O	O	O
Rarity	O	O	O	O	O
Amicability (friendliness/child safe)	O	O	O	O	O
Allergy-free	O	O	O	O	O
Activity level	O	O	O	O	O
Watchdog capability/protection	O	O	O	O	O
To save/rescue an animal in need	O	O	O	O	O
I had a similar/same dog before	O	O	O	O	O
Health (healthy breed/long life)	O	O	O	O	O
To breed dogs	O	O	O	O	O
To do dog sports/ work with the dog	O	O	O	O	O

How much did you think before you bought/adopted this dog? *

- 1.It was an impulsive decision (less than a day)
- 2.
- 3.
- 4.
- 5.I was thinking about it for years

How often do you take your dog for a walk? *

- 1.Never

- 2.
- 3.
- 4.
- 5.Very often (three or more times a day)

Do you actively play with your dog during walks or at home (e.g. fetching, tug-of-war, swimming, running)? *

- Yes
- No

How much do you play/exercise with your dog during walks? *

- 1.None, we just walk
- 2.
- 3.
- 4.
- 5.We regularly and vigorously play/exercise

Training of the dog*

Mark only one option

- None
- I trained it at home
- One course in dog school
- Regular dog school
- Private trainer
- Special training (e.g. sport or work related)

Did/do you regularly do sport/work activity with your dog? *

- Yes
- No

What age did you start to do sports/work with your dog? __________________

If you do not sport/work regularly with your dog anymore, when did you quit doing it (age)? __________________

How often/regularly do you work/sport with your dog – including the training occasions, too? *

- 1.Monthly or less
- 2.
- 3.
- 4.
- 5.Every day

What kind of sports/work do you do with your dog? Please list each sport in which you train/perform with your dog.___________________

Does your dog have qualifications, exams, competition placements etc.? *

Mark only one option

- Yes, several
- Yes, one or a few
- No

Dog's health

What is the body condition score of your dog? (please use the chart as guidance and pick the condition which is most fitting to your dog) *

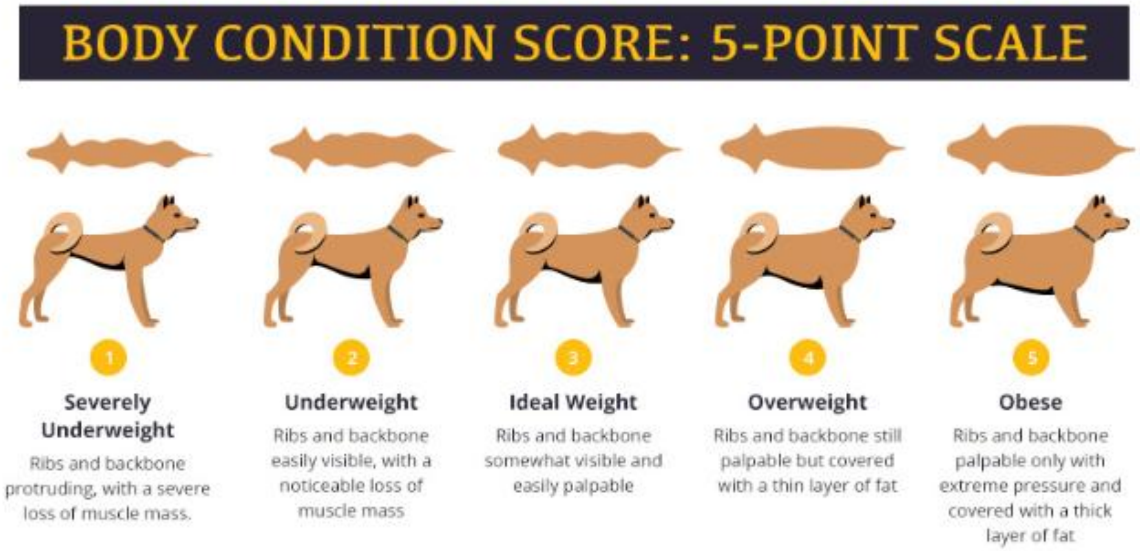

- Severely underweight
- Underweight
- Ideal weight
- Overweight
- Obese

Did the dog had any accident (leading to bone fracture, dislocation, a wound that required surgical repair)? *

- Yes
- No

Did your dog has any serious acute disease (infectious disease, stomach torsion etc.) *

- Yes
- No

Does your dog have ongoing (chronic) orthopedic condition (hip dysplasia, spinal illness etc.)? *

- Yes
- No

Does your dog have ongoing (chronic) neurological condition (epilepsy etc.)? *

- Yes
- No

Does your dog have ongoing (chronic) metabolic/ immunological condition (allergies, irritable bowel syndrome, diabetes, hormonal conditions etc.) ? *

- Yes
- No

Are there problems with the dog's vision and/or hearing? *

- Yes
- No

Did the mentioned problem permanently affect your dog's sporting/working ability or activity? *

- Yes
- No

Dog aging

Please tell about the occurence of the following phenomena: *

How often does your dog pace up and down, walk in circles and/or wander with no direction or purpose?	Never
How often does your dog stare blankly at the walls or floor?	Once a month
How often does your dog get stuck behind objects and is unable to get around?	Once a week

How often does your dog fail to recognize familiar people or pets?	Once a day
How often does your dog walk into walls or doors?	More than once a day
How often does your dog walk away while, or avoid, being petted?	

How often does your dog have difficulty finding food dropped on the floor? *

- Never
- 1-30% of times
- 31-60% of times
- 61-99% of times
- Always

Please select the most fitting option. If some of the listed behaviors do not occur in the case of your dog, please choose "the same" option. *

Compared with 6 months ago, does your dog now pace up and down, walk in circles and/or wander with no direction or purpose?	Much less
Compared with 6 months ago, does your dog now stare blankly at the walls or floor?	Slightly less
Compared with 6 months ago, does your dog urinate or defecate in an area it has previously kept clean?	The same
Compared with 6 months ago, does your dog have difficulty finding food dropped on the floor?	Slightly more
Compared with 6 months ago, does your dog fail to recognize familiar people or pets?	Much more
Compared with 6 months ago, is the amount of time your dog spends active?	
